# Phenotyping of α-1-Antitrypsin by liquid chromatography–high resolution mass spectrometry

**DOI:** 10.1016/j.clinms.2017.02.002

**Published:** 2017-02-20

**Authors:** Per Bengtson, Camilla Valtonen-André, Magnus Jonsson

**Affiliations:** a Department of Clinical Chemistry, University Health Care in Region Skåne, Lund, Sweden; b Department of Clinical Chemistry, University Health Care in Region Skåne, Malmö, Sweden

**Keywords:** P_i_ typing, LC–MS, Collision induced dissociation, Alpha 1-antitrypsin, SERPINA1, Isoelectric focusing

## Abstract

More than seventy-five isotypes of α-1-antitrypsin (AAT) have been described. To assess risks associated with AAT deficiency, isotype identification is necessary. Isoelectric focusing (IEF) is traditionally used for isotype differentiation, however, IEF has limited scope since it is a manual procedure that is not suitable for automation, and antitrypsin variants must differ in net charge in order to be resolved. In comparison, mass spectrometric assays are easily automated and offer a more complete solution for characterization of proteins. To capitalize on these advantages, we have developed a qualitative top-down liquid chromatography–mass spectrometry (LC–MS) method for selective phenotyping of AAT. This technique requires no sample pretreatment, and has the potential for use in routine clinical diagnostics. We have validated our LC–MS results against both DNA sequencing and IEF. Thus far, this method has identified the AAT variants P_Lowell_, S and Z, as well as unique fragments shared by different M alleles. Its high selectivity is indirectly illustrated by the detection of a variant carrying the amino acid substitution p.Ala308Ser, which cannot be visualized by IEF.

## Highlights

• A top-down LC–MS method for selective phenotyping of α-1-antitrypsin is presented.
• The method is automatable and requires no sample pretreatment.
• The most common alleles involved in antitrypsin deficiency are identified.
• The method represents a promising approach for phenotyping by LC–MS.

## 1 Introduction

For decades, phenotyping of α-1-antitrypsin (AAT) has been a cornerstone in the identification of hereditary AAT deficiency. The electrophoretic pattern representing AAT deficiency was first described in 1963 by Laurell and Eriksson [1], who also suggested its connection with degenerative pulmonary disease. Today, it is known that AAT deficiency is primarily associated with lung and liver disease [2].

AAT is an N-glycosylated serine protease inhibitor (serpin) that contains 394 amino acid residues in its secreted form [3]. The gene encoding AAT (*SERPINA1*) is located on chromosome 14 [4], [5], and expression occurs chiefly in hepatocytes, but also in monocytes and macrophages [2], [6]. Elastase is the primary target of AAT, and it has been suggested that this serpin plays a physiological role in protection of the lower respiratory tract against proteolytic destruction by human leukocyte elastase [7].

**Fig. 1:**
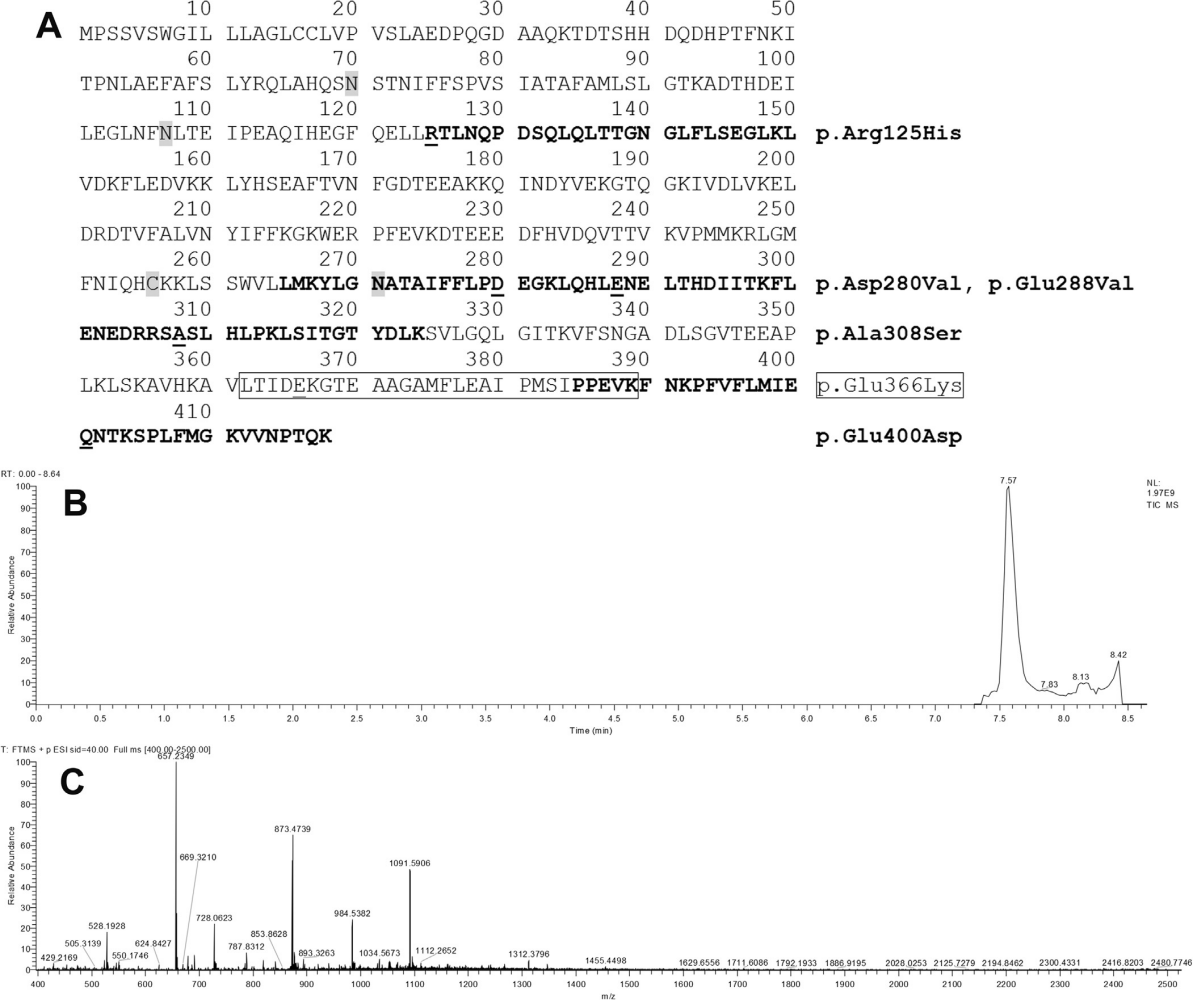
The complete amino acid sequence of AAT Uniprot P01009-1 shown with four fragment peptides (bold or boxed text) containing the p.ArgR125His, p.Asp280Val, p.Glu288Val, p.Ala308Ser, p.Glu366Lys and p.Glu400Asp shifts. The three glycosylation sites (N) and the cysteinylation site are grey-boxed. Typical results obtained using the LC–MS method: a total ion count (TIC) chromatogram (B); full scan spectra of chromatographic peak at 7.59 min (C).

Several alleles have been associated with decreased AAT levels in serum, and different alleles are associated with various clinical conditions [7]; thus, both the concentration and proteoform of AAT are of interest when diagnosing and managing patients. AAT proteoforms are traditionally named according to their proteinase inhibitor (P_i_) phenotype, which is visualized by the migration characteristics of the proteins in a pH 4–5 isoelectric focusing (IEF) gel [7], [8]. The most anodal and the most cathodal variants are assigned mobility letters from the beginning, and from the end, of the alphabet, respectively. Several normal alleles exist, most of which result in a protein, characterized via IEF as M mobility. The vast majority of these normal alleles code for variants termed as M1, M2, M3, and M4, exhibiting minor differences in migration via IEF. Accordingly, using IEF, it is difficult to distinguish these alleles, and they are often collectively referred to as M alleles. The S and Z variants are the two most commonly associated with decreased levels of S-AAT and disease. The allele encoding the S variant carries a missense mutation resulting in a glutamic-acid-to-valine substitution at codon 288 (*SERPINA1*:c.863A > T, p.Glu288Val), and the allele, encoding the Z variant, contains a missense mutation causing a glutamic-acid-to-lysine substitution at codon 366 (*SERPINA1*:c.1096G > A, p.Glu366Lys) (Fig. 1) [4], [9], [10], [11].

While IEF is a high resolution method with the ability to distinguish between small differences in isoelectric point, differentiating mutated variants from normal alleles requires a difference in charge states [12]. Furthermore, IEF requires several manual steps that are not easily automated. Mass spectrometry (MS) offers an easily automated analytical workflow combined with high specificity and is a promising tool for protein phenotyping. In 2011, Chen et al. [13] presented a liquid chromatography–tandem mass spectrometry (LC–MS/MS) method requiring pretreatment of the sample with trypsin digestion and addition of 13C/15N-labeled standard peptides that can be used to identify and quantitate S and Z variants, and validated their results versus IEF. Since standardized routine methods already exist for measuring AAT concentrations [14], we focused on developing a qualitative LC–MS method appropriate for selective phenotyping of AAT, with no requirements for pretreatment of samples. We validated this method versus both IEF and DNA sequencing.

## 2 Methods

### 2.1 Samples/study population

Serum samples (n = 84) were obtained from individuals referred to the Region Skåne Clinical Chemistry Laboratory for evaluation of possible AAT deficiency. All samples were submitted for AAT quantification and phenotyping. For 18 of the 84 subjects, an EDTA whole blood serum sample was also included in the study. All samples were anonymized and stored frozen at −20 °C, for no more than 6 months prior to analysis.

### 2.2 Phenotyping AAT variants by isoelectric focusing (P_i_ typing)

Cysteine-reduced serum was separated by IEF on polyacrylamide gel as described by Jeppsson and Franzén [12]. An experienced interpreter deduced the P_i_ type from the resulting pattern.

### 2.3 LC–MS analysis

We performed high-resolution 2D LC–MS as described in the online Supplemental Materials and Methods. Briefly, serum was diluted 1:10 with PBS, and 15 µL of the diluted sample was injected into the 2D LC–MS system, which consisted of an in-line anti-AAT affinity column and an analytical C4 column coupled to a hybrid quadrupole-orbitrap mass spectrometer. The purified AAT was fragmented using in-source collision-induced dissociation (CID). Full scan mode on the mass spectrometer was set to 140,000 at *m*/*z* 200, its highest resolution. Unique fragments covering selected amino acid sequences were identified by comparing AAT serum samples from AAT-genotyped and P_i_-typed individuals.

### 2.4 Calculations

Peak intensities in isotope distributions of selected fragments were filtered, summed and plotted using R software (R version 3.3.1, a language and environment for statistical computing, R Foundation for Statistical Computing, Vienna, Austria. https://www.R-project.org/). Mann-Whitney U statistics were also calculated using R.

### 2.5 Genotyping

Genotyping was performed as described in the Supplemental Materials and Methods. Briefly, DNA was isolated from whole blood and then subjected to PCR amplification and Sanger sequencing. The analyzed amplicons encompassed all exons of *SERPINA1*, including exon/intron borders and the 5′- and 3′-untranslated regions (for primers, see Table S1). The reference sequences used for primer design and subsequent interpretation of results were NG_008290, NM_001002236.2, and NP_001002236.1

## 3 Results and discussion

### 3.1 AAT variants phenotyped by LC–MS

Eighteen samples, with known genotypes (sequence data) and P_i_ type (IEF data) (Table S2), were used to identify and characterize unique fragments based on selected amino acid shifts (Table 1). Amino acid substitutions caused the variant fragments to differ sufficiently in mass from the wild-type to allow discrete identification using MS alone. The locations of these fragments in the wild-type reference amino acid sequence of AAT (Uniprot P01009-1) are shown in Fig. 1. Unique fragments were named according to the amino acid position and shift of the variant (*e.g.*, 288Val for the p.Glu288Val variant and 288Glu(wt) for the corresponding wild-type variant) (Table 1). Fig. 1 also presents a representative total ion count chromatogram and a full scan mass spectrum.

**Table 1:** MS peptide fragments used for phenotyping.

Amino acid shift position	Fragment name	Mean experimental monoisotopic mass (Da) a	Theoretical monoisotopic mass (Da)	Difference (Da) b	Peptide fragment position	Peptide fragment sequence (verified by DNA sequencing) c	Charge state on fragment used for measurements	Isotopologues of fragment used for intensity measurements
125	125Arg(wt)	2102.080	2102.076	<0.01	125–143	(L)RTLNQPDSQLQLTTGNGLF(L)	5	2–4 additional neutrons
	125His (Pi M2,M4) d	2083.042	2083.033	<0.01		(L)HTLNQPDSQLQLTTGNGLF(L)	5	2–4 additional neutrons
280	280Glu(wt)	6983.684	6983.653	<0.04	265–325	(L)LMKYLGNATAIFFLPDEGKL QHLENELTHDIITKFLENED RRSASLHLPKLSITGTYDLK S(V)	7	1–4 additional neutrons
	280 Val (Pi P _Lowell_) d	6967.754	6967.715	<0.04		(L)LMKYLGNATAIFFLPDVGKL QHLENELTHDIITKFLENED RRSASLHLPKLSITGTYDLK S(V)	7	1–4 additional neutrons
288	288Glu(wt)	6983.684	6983.653	<0.04	265–325	(L)LMKYLGNATAIFFLPDEGKL QHLENELTHDIITKFLENED RRSASLHLPKLSITGTYDLK S(V)	7	1–4 additional neutrons
	288Val (Pi S) d	6953.715	6953.679	<0.04		(L)LMKYLGNATAIFFLPDEGKL QHLVNELTHDIITKFLENED RRSASLHLPKLSITGTYDLK S(V)	7	1–4 additional neutrons
308	308Ala(wt)	6983.684	6983.653	<0.04	265–325	(L)LMKYLGNATAIFFLPDEGKL QHLENELTHDIITKFLENED RRSASLHLPKLSITGTYDLK S(V)	7	1–4 additional neutrons
	308Ser	6999.670	6999.648	<0.03		(L)LMKYLGNATAIFFLPDEGKL QHLENELTHDIITKFLENED RRSSSLHLPKLSITGTYDLK S(V)	7	1–4 additional neutrons
366	366Glu(wt)	2957.605	2957.513	<0.10	362–389	(V)LTIDEKGTEAAGAMFLEAIP MSIPPEVK(F)	4	1–4 additional neutrons
	366Lys (Pi Z) d	2956.664	2956.565	<0.10		(V)LTIDKKGTEAAGAMFLEAIP MSIPPEVK(F)	4	1–4 additional neutrons
400	400Glu(wt)	3932.126	3932.110	<0.02	385–418	(I)PPEVKFNKPFVFLMIEQNTKSPLFMGKVVNPTQK	5	1–3 additional neutrons
	400Asp (Pi M3,M2) d	3918.114	3918.094	<0.02		(I)PPEVKFNKPFVFLMIDQNTKSPLFMGKVVNPTQK	5	1–3 additional neutrons

a Mean values based on multiple measurements performed on one or several of the 18 sequenced samples.

b Difference between mean experimental monoisotopic mass and theoretical monoisotopic mass.

c Amino acid shifts are underlined. The reference amino acid sequence is Uniprot P01009-1, including the 24 amino acid leader sequences.

d Pi-type associated with given amino acid shift.

**Fig. 2:**
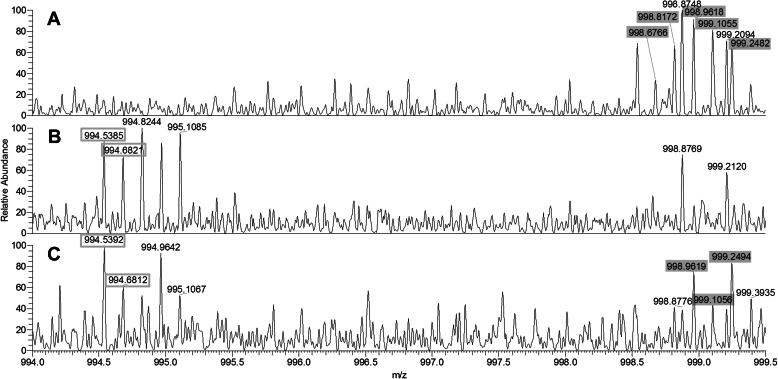
Mass spectra in the range *m*/*z* 994-999.5 of 288Glu(wt) homozygous (A), 288Val homozygous (B), 288Glu(wt)/288Val heterozygous (C) samples. Selected mass numbers unique for 288Glu(wt) (filled boxes) and for 288Val (empty boxes) are indicated.

The amino acid shift *SERPINA1*:p.Glu288Val (c.863A > T) is characteristic for P_i_ S. With a size of nearly 7 kDa, the fragment containing the amino acid position 288 proved to be the largest of the studied fragments (Table 1). The most significant results for differentiating individuals genotyped to carry p.Glu288Val from those carrying wild-types arose from peaks at charge state 7 (Fig. 2A–C). As shown in Fig.2A, the resolving power of the mass spectrometer was capable of discriminating the isotopes of the protein fragment, 288Glu(wt), starting with the monoisotopic peak at *m*/*z* 998.6766 followed by 998.8172, 998.9618, 999.1055, and so forth. The theoretical mass shift caused by p.Glu288Val is ∼−29.9742 Da, which, at charge state 7, results in a loss of *m*/*z* ∼4.28 leading to the detection of 288Val-specific peaks at *m*/*z* 994.5385, 994.6821 and so forth, in samples homozygous for p.Glu288Val (Fig.2B). Fragments 288Glu(wt) and 288Val could be used in a predictable and reproducible manner to detect the presence of p.Glu288Val. In contrast to the subjects who were homozygous for p.Glu288Val, the individuals who were heterozygous displayed both 288Val and 288Glu(wt) (Fig.2C). The difference between experimental and theoretical masses for this fragment was <0.04 Da (Table 1).

**Fig. 3:**
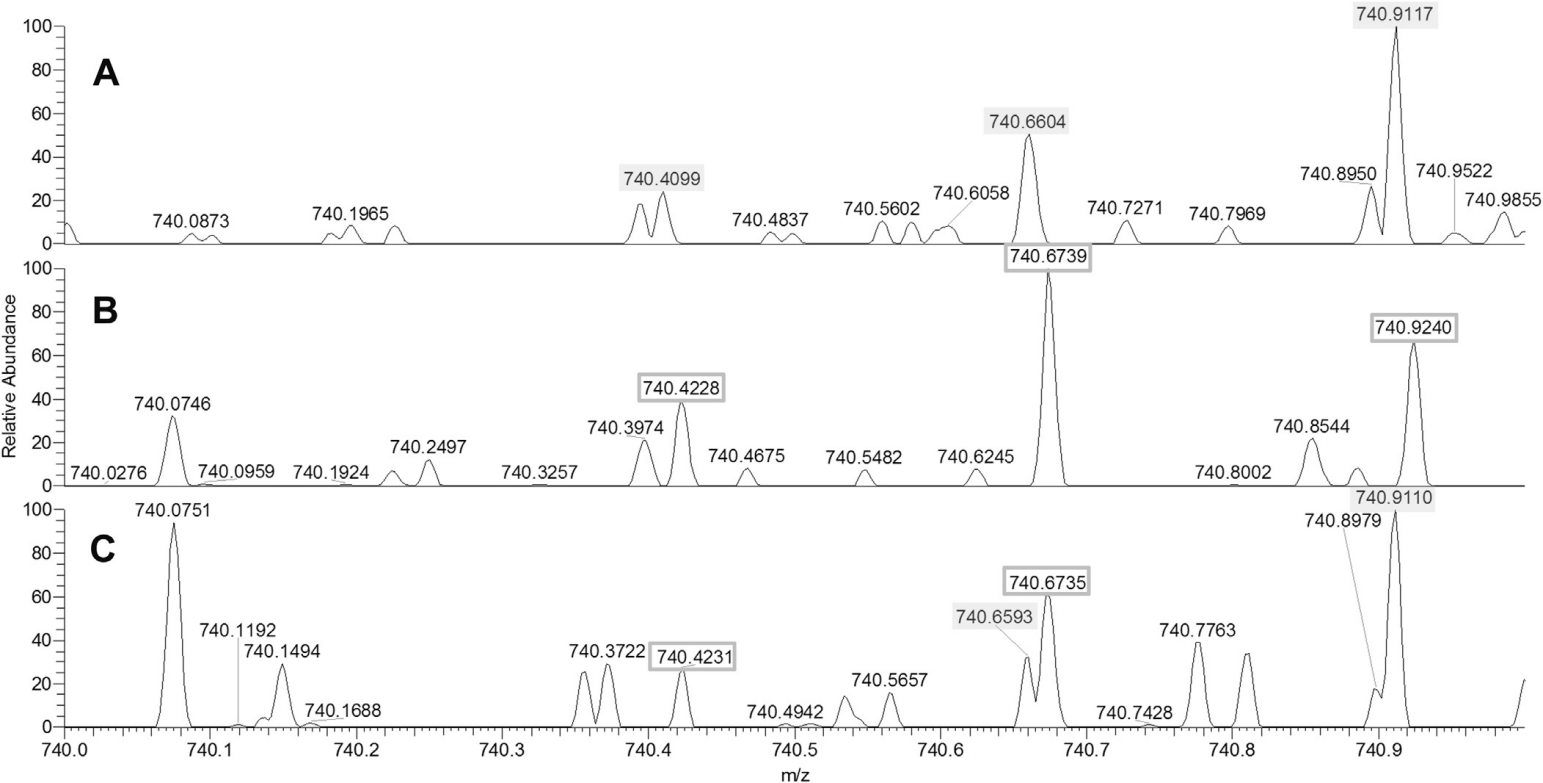
Mass spectra in the range *m*/*z* 740–741 of 366Glu(wt) homozygous (A), 366Lys homozygous (B), and 366 Glu(wt)/366Lys heterozygous (C) samples. Selected mass numbers unique for 366Glu(wt)(filled boxes) and for 366Lys (empty boxes) are indicated.

The amino acid shift *SERPINA1*:p.Glu366Lys (*SERPINA1*:c.1096G > A) is characteristic for P_i_ Z. The mass shift caused by p.Glu366Lys is 0.94763 Da. In samples confirmed by genotyping to contain the p.Glu366Lys shift, significant fragments containing amino acid 366 were found at around *m*/*z* 740 and charge state 4, exemplified by the experimental results at 740.6604 for 366Glu(wt) and 740.4228 for 366Lys shown in Fig. 3(A and B). The mass spectrometer was capable of resolving nearly overlapping isotopic peaks, as exemplified by 740.6604 in Fig.3A and 740.6593 in Fig.3C for 366Glu(wt), and 740.6739 in Fig.3B and 740.6735 in Fig3C for 366Lys. Homozygous and heterozygous samples could, therefore, be phenotyped in the same manner as previously described for p.Glu288Val.

Fragments for the amino acid shifts p.Glu400Asp (in the M2- and M3-alleles), p.Asp280Val (in P Lowell-allele), p.Ala308Ser (rs 141620200) and p.Arg125His (in the M2- and M4-alleles) were discovered and scrutinized in the same manner as described for p.Glu288Val and p.Glu366Lys, see Supplemental data (Figs. S1–S4).

### 3.2 Performance characteristics

The observed agreement between the theoretical masses (based on DNA sequencing results) and the experimental masses and mass shifts (based on LC–MS results), detailed in Table 1, supports our conclusion that the analyzed fragments consist of the amino acid sequences presented in Fig. 1 and that the method has a high degree of selectivity. To further validate the method, we analyzed sixty-six additional serum samples by IEF and LC–MS. These samples contained Z, S, and M alleles, and manual evaluation of the mass spectrometric results identified them correctly (Table S3).

**Fig. 4:**
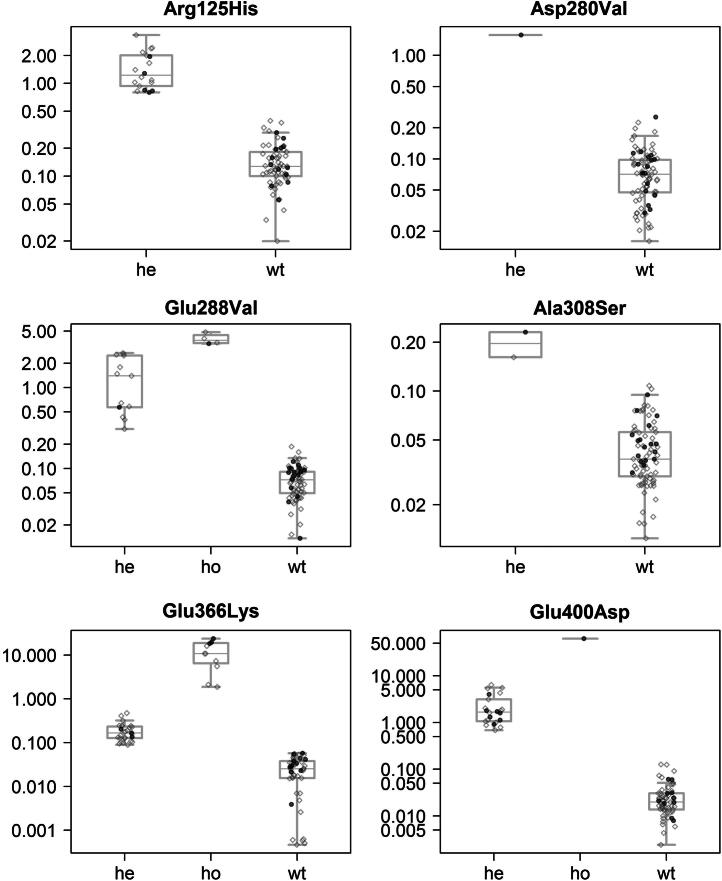
Dot plots of ratios between indicated intensities for non-wild-type fragments and wild-type fragments. The ratios are plotted against categorized heterozygous (he), homozygous (ho) and wild-type (wt) samples. Dark grey circles represent DNA-sequenced samples and light grey diamonds represents non-sequenced samples. Overlaid are box plots with further information. The box represents the first and third quartiles, the band inside the box indicates the median and the whiskers indicate the lowest or highest ratio still within 1.5 times interquartile range of the lower or higher quartile.

For a more rapid, objective and automated evaluation of the raw data produced by the LC–MS method, we programmed the software R, a language and environment for statistical computing, to filter and sum the intensities in the corresponding isotope distributions. For each sample, the ratio between the summed intensities for variant and wild-type fragments was plotted against P_i_ type/genotype (Fig. 4). The populations of ratios for heterozygous samples were tested against ratios for corresponding populations of wild-type or homozygous mutated genotype samples using the Mann–Whitney *U* test. The p-values were <0.000005 for all but two tests, indicating a strong significance for a true difference in ratios. The p-value for heterozygous p.Glu288Val versus homozygous was <0.0005. With only one sample homozygous for p.Glu400Asp the result for Mann-U-Whitney test of heterozygous p.Glu400Asp versus homozygous still reached a p-value of <0.05.

Fig. 4 and the statistical calculations suggest that the presented method has adequate sensitivity and selectivity to discriminate between wild-type homozygous, heterozygous and variant homozygous samples for all variants investigated. Of those, the Z-allele is associated with the lowest protein expression (∼15% of normal). It was encouraging to note that all twenty-three samples heterozygous for the Z-allele were identified from the homozygous state by the ratio between the summed intensities for variant and wild-type fragments. Seven of these heterozygous samples were compound heterozygous for SZ.

Daily external spectral mass calibration of the instrument and the use of lock mass ensured low deviations in instrument performance. Imprecision of mass numbers for the studied wild-type fragments were calculated based on data from repeated analysis of patient samples (at least eight measurements per chromatographic peak). Coefficients of variation (CV) for mass number within day/between day were 0.00020%/0.00024% for 125Arg(wt), 0.00020%/0.00022% for 288Glu(wt), 0.00016%/0.00018% for 366Glu(wt), and 0.00006%/0.00006% for 400Glu(wt). The low CV levels demonstrate that the method is highly selective and minimizes the risk of overlapping of unique fragment mass numbers.

Thawed samples stored at 4 °C (n = 20) for up to 7 days could be phenotyped without any significant drop in signal-to-noise ratio (data not shown).

### 3.3 Fragmentation technique

LC–MS has been used previously in our laboratory for phenotyping intact proteoforms [15], [16], [17], [18], although the possibility of phenotyping intact AAT was ruled out early on during method development. One hurdle that was not easily overcome was the high complexity of the *m*/*z* charge distribution, which could be largely explained by the three highly diverse N-linked glycoconjugates carried by AAT [19]. Although the mass spectrometer did eventually provide data on intact AAT proteoforms, reproducible results with sufficiently high resolution were not obtained, and, accordingly, we were unable to robustly resolve the mass differences of approximately 1 Da between the p.Glu288Val and p.Glu366Lys alleles (data not shown). Targeted top-down fragmentation techniques have been utilized by other researchers to differentiate proteoforms [20]. In the Thermo Q Exactive instrument that was used, the major fragmentation technique is higher-energy collisional dissociation (HCD). Due to the above-mentioned complex charge distribution of intact AAT, substantial loss of signal in the HCD cell, and the relatively low expression of AAT protein from the p.Glu366Lys allele, we opted to apply a non-targeted fragmentation technique, in-source collision-induced dissociation (CID), instead. With this method, ions from the source entering the vacuum region are accelerated, prompting collisions with surrounding species that eventually produce sufficient energy to yield fragment ions. Inasmuch as precursor ions cannot be selected and isolated with in-source CID, the heredity of the product ions is lost. However, in our case, in-source CID achieved a significant and crucial increase in sensitivity over the HCD fragmentation technique, which, together with the selectivity provided by the affinity column, provided unique and reproducible fragments.

In-source CID tended to predominantly produce terminal fragments. Therefore, it was not surprising that peptide fragments unique for p.Glu400Asp, located near the C terminus, gave the strongest signals of the studied amino acid shifts (Fig. S1). The fragment including the p.Arg125His amino acid shift is located in the more central region of the protein, between two N-linked glycoconjugates, which might explain the weaker signals (Fig. S4). Using the described settings, we were unable to find unique fragments for M1A (Val237Ala). Unfortunately, no sample P_i_ phenotyped as F (*SERPINA1*:p.Arg247Cys (c.739C > T)) was available for genotyping. Given that the p.Arg247Cys amino acid shift is located only 10 amino acids from the M1A determinant Val237, it appears that this shift might be a challenge for the fragmentation method applied in the current setup. Supplementary fragmentation techniques such as electron transfer dissociation could aid production of alternative and complementary fragments in the more central parts of AAT. Glycopeptides tend to give weak signals in mass spectrometry, which is one of the factors hindering greater amino acid coverage via the tryptic digests in bottom-up LC–MS methods. CID can be used for partial or complete release/removal of glycoconjugates from the amino acid backbone [21]. This was an effective approach for the fragment 265–325 comprising p.Asp280Val, p.Glu288Val, and p.Ala308Ser, in which position Asn271 is a documented glycosylation site (Uniprot P01009). With the specified LC–MS settings, fragment 265–325 is robustly detected without its glycoconjugate.

### 3.4 LC–MS versus IEF

When phenotyping is performed using IEF, the quality assessment of the run and phenotype determination require human visual inspection. As a consequence, the reported P_i_ type is dependent upon the subjective inferences of the interpreter. The alternative analytical method proposed here is combination of LC–MS and R analysis, which offers a workflow that is amenable to procedural automation and standardized data evaluation. The ability to define statistical limits for run approval, and ratios to identify a phenotype, provides objective metrics for run interpretation. When optimized, this translates to more consistent and reliable data output, and, hence, a greater quality of analysis. There are disadvantages with the LC–MS method. For example, using in-source CID, precursor ions cannot be selected and isolated, hence, the information correlating fragment ions with their precursor ions is lost in much the same way as occurs with bottom-up techniques.

## 4 Conclusions

The presented top-down LC–MS method requires no sample pretreatment and is capable of identifying two of the most common and important AAT alleles involved in antitrypsin deficiency (Z and S), as well as P_Lowell_, and several M alleles. The described method has the potential to identify other amino acid shifts not studied here, although each variant allele would require thorough validation. As a proof of concept, we believe the method presented here represents a promising approach for AAT-phenotyping by LC–MS.

## Funding

This research did not receive any specific Grant from funding agencies in the public, commercial, or not-for-profit sectors.

## Conflict of interest

The authors wish to confirm that there are no known conflicts of interest associated with this publication and there has been no significant financial support for this work that could have influenced its outcome.
