# Supplementary material for: Phenotyping of α-1-Antitrypsin by liquid chromatography–high resolution mass spectrometry
Source: Clin Mass Spectrom. 2017 Feb 20;2:34–40. doi: 10.1016/j.clinms.2017.02.002 (PMC11324609; doi:10.1016/j.clinms.2017.02.002)
Supplement: Supplementary data [file mmc1.docx]

# Supplemental Materials and Methods

## LC-MS

### Reagents

Reagents and their sources were as follows: research-grade acetonitrile (MeCN), glycine, phosphate-buffered saline (PBS), and purified AAT were from Sigma-Aldrich; formic acid was from Fluka; polyclonal rabbit anti-human AAT was from Agilent (Dako A0012); POROS-aldehyde self-pack medium (1-6028-02) and positive external calibration solution (88323) were from Thermo Scientific. All other chemicals and solvents were of the highest analytical LC-MS grade available from commercial sources and were used without further purification.

### Immunoaffinity column preparation

Polyclonal anti-AAT antibodies were conjugated to POROS-aldehyde self-pack medium according to the manufacturer’s instructions. The antibodies (1 mg) were coupled to 0.1 g of medium, and, thereafter, the antibody conjugated medium was stored in PBS containing 0.01% (v/v) sodium azide at 4 °C. The antibody conjugated medium was packed in empty 20 x 1 mm pre-columns from Upchurch.

### Identification of relevant fragmented AAT peptides

Analysis of the AAT was performed on a Thermo Fisher Q Exactive MS with the standard heated electrospray source (HESI) controlled by Xcaliber software (ver. 3.0.63) operated in positive ion mode. The MS was interfaced to a 2D-LC and an autosampler system (Thermo Fisher). The autosampler loaded 15 µL of the diluted serum onto the first LC dimension (i.e., the affinity column described above). The affinity-purified fraction was introduced into the second LC dimension consisting of a 50 x 1 mm i.d. monolith column (Proswift RP-4H, Thermo Fisher). Mobile phase A consisted of PBS; mobile phase B was 100 mM glycine with 2% formic acid (by volume); mobile phase C was water:acetonitrile:formic acid (98:2:1 by volume); and mobile phase D was acetonitrile:water:formic acid (98:2:1 by volume). Mobile phases A and B were used for the affinity column, and phases C and D were applied for the analytical column. The LC-MS method took a total of 10 min to run at a flow rate of 200 µL/min.

The Q Exactive was operated in a full scan mode. Direct infusion of purified wild-type AAT was performed to tune the instrument and optimize the CID fragmentation parameters. Optimal ESI conditions were as follows: spray voltage, 4.5 kV; capillary temperature, 350 ˚C; sheath gas pressure, 35 L/hr; auxillary gas, 10; probe heater temperature 300 ˚C; S-Lens RF-level, 50. MS parameters were CID 40–45 and micro scan count 2–4 with a resolution setting of 140,000 at m/z 200. The mass spectrometer was calibrated with an external calibrator on a daily basis. The dominant, and always present, AAT-endogenous glycoconjugate fragment, [N-acetyl neuraminic acid-galactose-N-acetylglucosamine + H]^+^ at m/z 657.2349, was used as lock mass.

For establishment of specific fragments the MS spectra were analyzed manually, with help from the web tool FindPept (Expasy, SIB Swiss Institute of Bioinformatics) and Xtract software (Thermo Fisher) or automatically using ProSight PC 3.0 in single-protein mode (Thermo Fisher). Variable modifications included methionine oxidation, N-acetylation of the N terminus, cysteinylation, N-glycosylation, phosphorylation, and alkylation of cysteines. Uniprot P01009-1 was used as reference protein sequence.

**Genotyping**

The isolation of genomic DNA from EDTA whole blood was done using an Agentcourt Genfind v2 blood and serum genomic DNA isolation kit (Beckman Coulter), and the procedure was performed on a Biomek FXP robot (Beckman Coulter).

Primers for amplicon generation were designed to span all exon/intron borders, also including the 5` and 3`UTR regions. The reference sequences used for primer design and subsequent interpretation of results were NG_008290, NM_001002236.2, and NP_001002236.1. The primary PCR reactions were carried out using primers with M13 tails (DNA Technology, Denmark) (Supplemental Table 1). The PCR was performed with a BigDye Direct Cycle Sequencing Kit (Applied Biosystems) essentially according to the manufacturer’s instructions, with a total DNA concentration of 0.5 ng/μL in a reaction volume of 10 μL. The reactions were carried out on an ABI Gene Amp 9700 (Applied Biosystems) with a protocol comprising 5 min initialization at 96 ˚C followed by 35 cycles consisting of 30 s at 94 ˚C, 45 s at 62 ˚C and 45 s at 68 ˚C. At the end of the program, there was a 2-min incubation at 72 ˚C.

The sequence reactions were performed using a BigDye Direct Cycle Sequencing Kit, (Applied Biosystems) with 10 μL of PCR-product added to a total reaction volume of 13 μL. The PCR protocol consisted of 15 min at 37 ˚C, 2 min at 80 ˚C, and 1 min at 96 ˚C, followed by 25 cycles of 10 s at 96 ˚C, 5 s at 50 ˚C, and 4 min at 60 ˚C. Thereafter, the sequence products were purified using a Big Dye XTerminator Purification Kit (Applied Biosystems) and subsequently subjected to DNA sequencing on an Applied Biosystem 3500 Genetic Analyzer.

Sequence data were analyzed using SeqScape 2 (Applied Biosystems), and the data on each individual sample were compared with the chosen reference sequences.

## Supplemental Figures


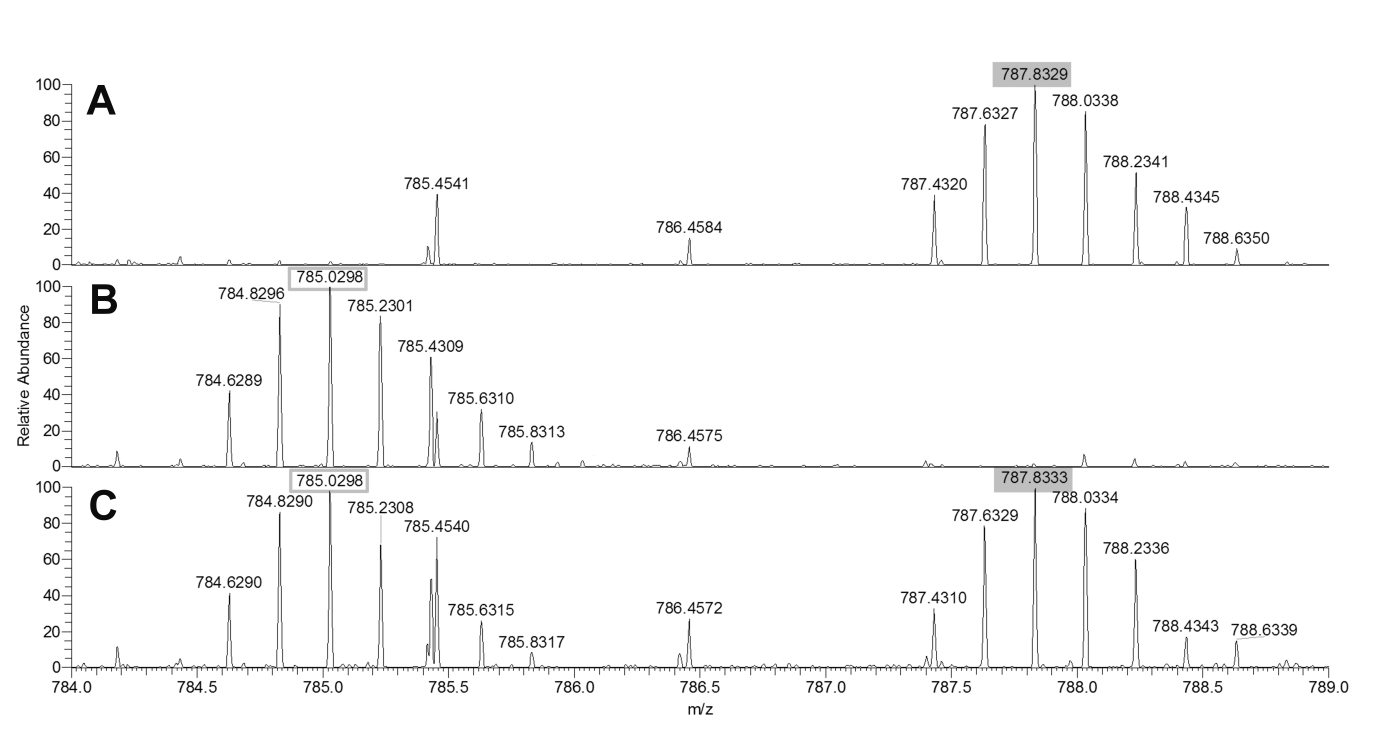


**Figure S1. p.Glu400Asp**

Mass spectra in the range 784–789 m/z of 400Glu(wt) homozygous (A), 400Asp homozygous (B), and 400Glu(wt) /400Asp heterozygous (C) samples detecting specific fragments and their isotope distribution at charge state 5. Mass numbers with the strongest signal unique for 400Glu(wt) (filled boxes) and mass numbers for 400Asp (empty boxes) are indicated.


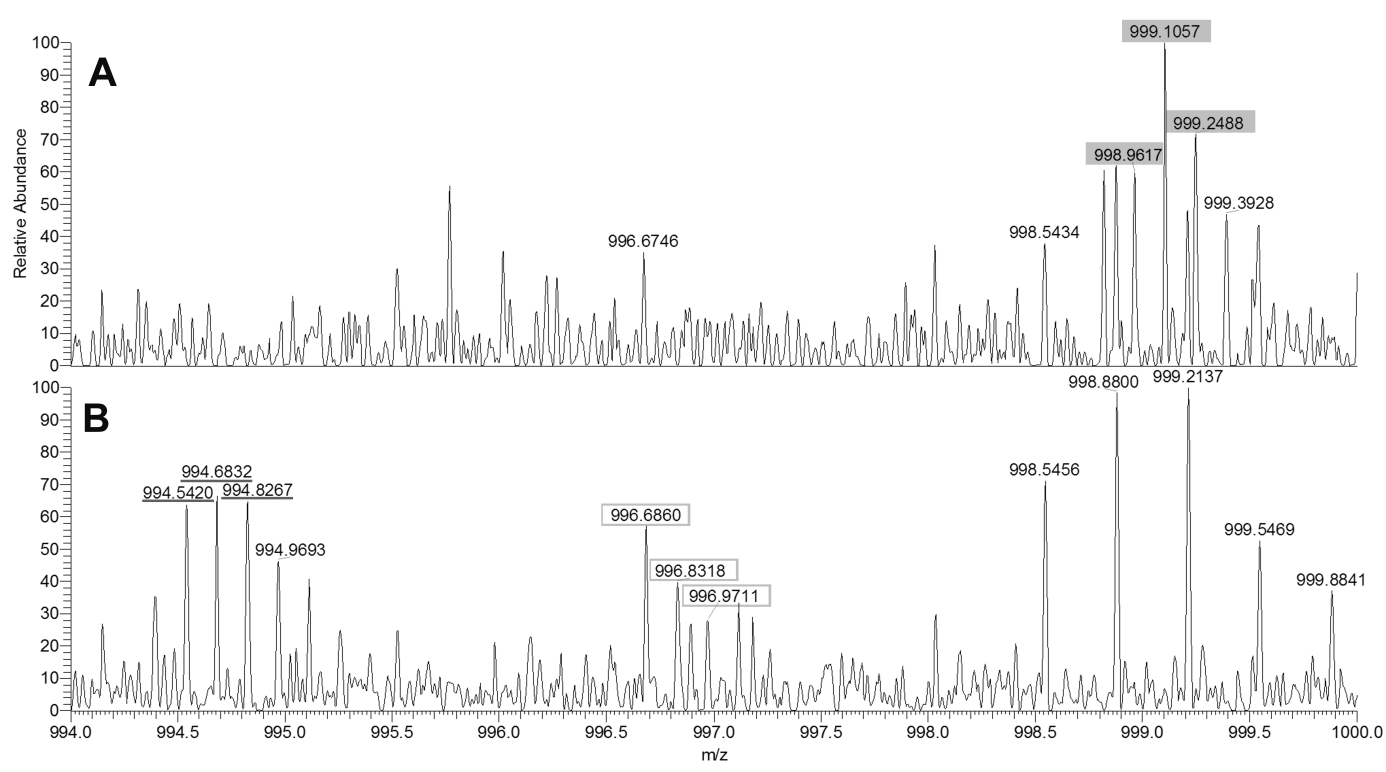


**Figure S2. p.Asp280Val**

Mass spectra in the range 994–1000 m/z of 280Asp(wt) homozygous (A) and the 280Val/288Val heterozygous (B) sample detecting specific fragments and their isotope distribution at charge state 7. Selected mass numbers unique for 280Asp(wt) (filled boxes), fragments unique for 280Val (empty boxes), and mass numbers unique for 288Val (underlined) are indicated.


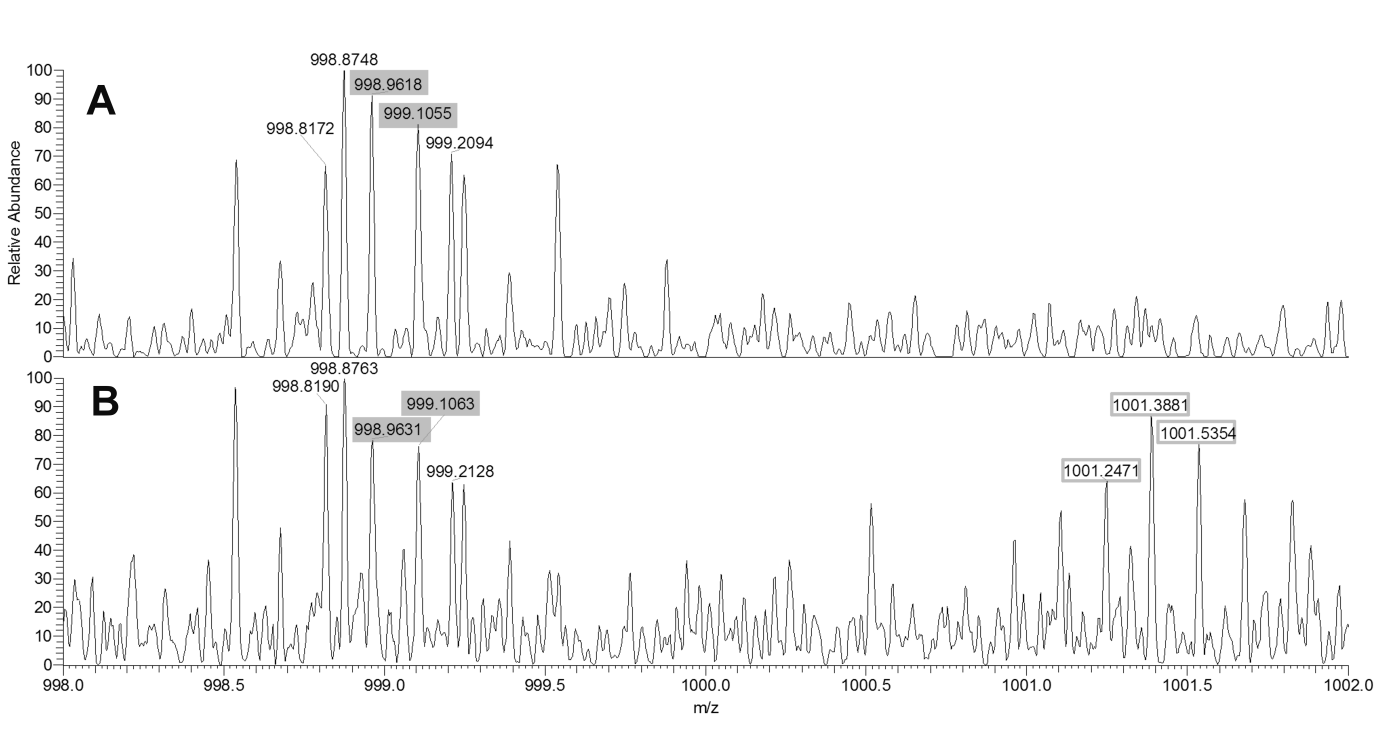
**Figure S3. p.Ala308Ser**

Mass spectra in the range 998–1002 m/z of 308Ala(wt) homozygous (A) and the 308Ala(wt)/308Ser heterozygous (B) sample detecting specific fragments and their isotope distribution at charge state 7. Selected mass numbers unique for 308Ala(wt) (filled boxes) and for 308Ser (empty boxes) are indicated.


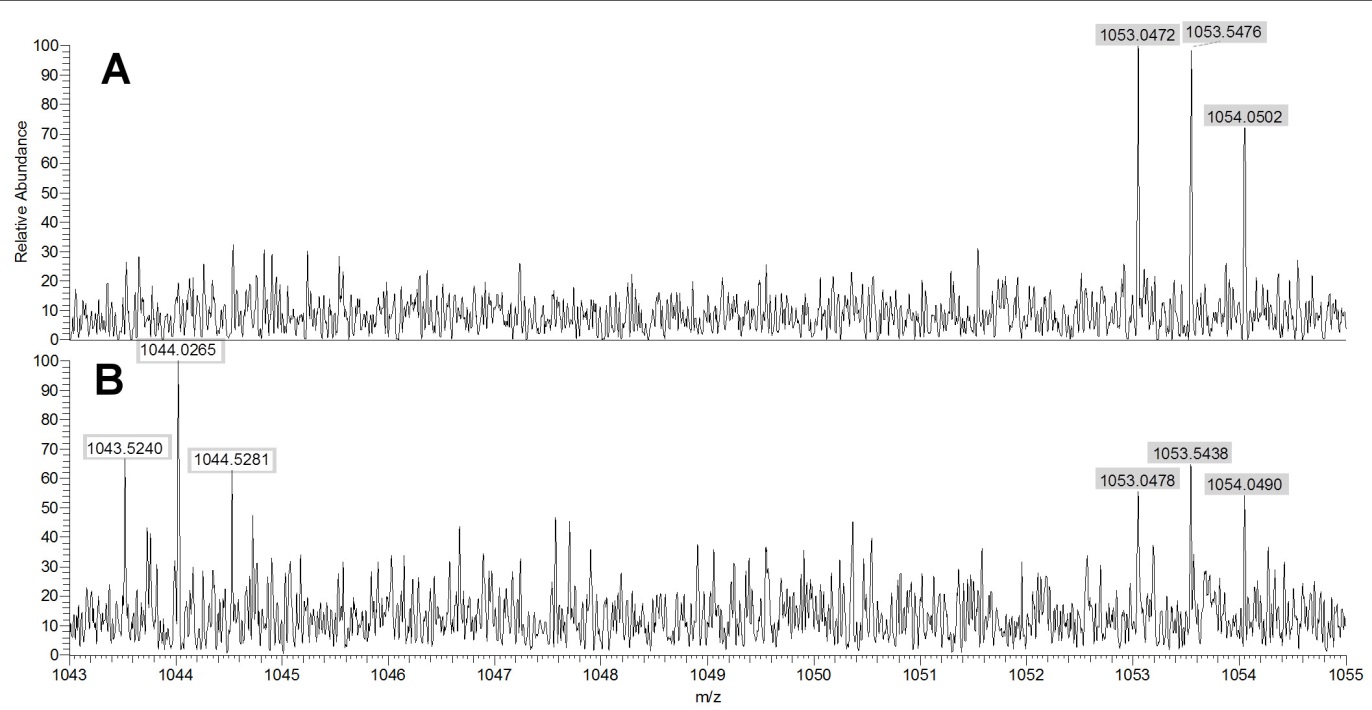


**Figure S4. p.Arg125His**

Mass spectra in the range 1043–1054 m/z of 125Arg(wt) homozygous (A) and 125Arg(wt) /125His heterozygous (B) samples detecting specific fragments and their isotope distribution at charge state 2. Mass numbers unique for 125Arg(wt) (filled boxes) and for 125His (empty boxes) are indicated.

## Supplemental Table 1.

Primers used for PCR amplification. Upper and lower case letters indicate the gene-specific sequences and M13 tails, respectively. The exons are systematically numbered (NM_001002236.2).

| Exon | Forward primer | Reverse primer |
| --- | --- | --- |
| Exon 1 | 5´ tgtaaaacgacggccagtGTGTCTGCCGGGCAATGAGCA 3´ | 5´ caggaaacagctatgaccCCGCTGCTCTACATCCACTCAC 3´ |
| Exon 2+3 | 5´ tgtaaaacgacggccagtGCCTGACGGTTGTGTCTGCCT 3´ | 5´ caggaaacagctatgaccCCTCCTGCCCCAGACCTGCT 3´ |
| Exon 4 | 5´ tgtaaaacgacggccagtACGTGGTGTCAATCCCTGATCACTG 3´ | 5´ caggaaacagctatgaccCTGTCTGGCTGGTTGAGGGT 3´ |
| Exon 4 | 5´ tgtaaaacgacggccagtTCTCCCCAGTGAGCATCGCTA 3´ | 5´ caggaaacagctatgaccGGCCCATAATGCATTGCCAAGGAGA 3´ |
| Exon 5 | 5´ tgtaaaacgacggccagtCTTCCAAACCTTCACTCACCCCTGGT 3´ | 5´ caggaaacagctatgaccTCCTCATGGAGCATGGATGGCG 3´ |
| Exon 6 | 5´ tgtaaaacgacggccagtTGTGGGTGGAGGACACAGGAGTA 3´ | 5´ caggaaacagctatgaccAGCTGCAGCCCCCACACATTC 3´ |
| Exon 7 | 5´ tgtaaaacgacggccagtTGGACAGAGGGGAGCCAGC 3´ | 5´ caggaaacagctatgaccCAGAGAAAACATGGGAGGGATTACA 3´ |
| Exon 7 | 5´ tgtaaaacgacggccagtAGTCTCCCCTCTTCATGGGAA 3´ | 5´ caggaaacagctatgaccCGGGTCAAGAGGAGGACATTG 3´ |
| Exon 7 | 5´ tgtaaaacgacggccagtAGAACTGCCTGATCGTGCC 3´ | 5´ caggaaacagctatgaccACCACAGGGGCTATTCAGGA 3´ |
| Exon 7 | 5´ tgtaaaacgacggccagtGCAGCCTGACTTCTTTGTGC 3´ | 5´ caggaaacagctatgaccATCCTAGGGGGCTTGGTGAT 3´ |
| Exon 7 | 5´ tgtaaaacgacggccagtACCGACTGTTTCCCACACAA 3´ | 5´ caggaaacagctatgaccGAAGGGGAGAGTGTGGTGAG 3´ |

## Supplemental Table 2.

Correlation between genotyping, phenotyping by IEF or phenotyping by LC-MS. 18/18 samples were concordant.

| **#** | **Genotyping** | | | | | **IEF** | **LCMS** | | | | | |
| --- | --- | --- | --- | --- | --- | --- | --- | --- | --- | --- | --- | --- |
|  |  |  |  |  |  |  | **Deviations from wt-aa sequence mass** | | | | | |
|  | **Contig pos** | **HGVS** | **Amino acid change** | **Ref.** | **Result** | **Result** | **p.Arg125His** | **p.Glu280Val** | **p.Glu288Val** | **p.Ala308Ser** | **p.Glu366Lys (PiZ)** | **p.Glu400Asp (PiM2,M3)** |
|  | **SeqScape** | **position** |  |  |  |  | **(PiM2,M4)** | **(PiL_Lowell_)** | **(PiS)** |  |  |  |
| 1 | Exon 1a | c.-409G>A |  | rs56367058 | G/A | MM |  |  |  |  |  | 400Asp/400Glu(wt) |
|  | Exon 3 | c.424C>T |  | rs20546 | C/T |  |  |  |  |  |  |  |
|  | Exon 6 | c.1200A>C | GAA>GAC p.Glu400Asp | rs1303 | A/C |  |  |  |  |  |  |  |
|  | 3' UTR | c.*224G>A |  |  | G/A |  |  |  |  |  |  |  |
|  | 3' UTR | c*854T>C |  | rs1051052 | T/C |  |  |  |  |  |  |  |
|  | 3' UTR | c.*968T>C |  | rs1243166 | C/C |  |  |  |  |  |  |  |
|  | 3' UTR | c.*1067G>A |  | rs11628917 | G/A |  |  |  |  |  |  |  |
|  | 3' UTR | c.*1221A>G |  | rs11832 | A/G |  |  |  |  |  |  |  |
| 2 | Exon 1a | c.-535G>C |  | rs8008743 | C/T | MM | 125His/125Arg(wt) |  |  |  |  | 400Asp/400Asp |
|  | Exon 1a | c.-439C>T |  | rs8004738 | T/T |  |  |  |  |  |  |  |
|  | Exon 3 | c.374G>A | CGT>CAT p.Arg125His | rs709932 | G/A |  |  |  |  |  |  |  |
|  | Exon 6 | c.1200A>C | GAA>GAC p.Glu400Asp | rs1303 | C/C |  |  |  |  |  |  |  |
|  | 3' UTR | c.*224G>A |  |  | A/A |  |  |  |  |  |  |  |
|  | 3' UTR | c.*968T>C |  | rs1243166 | T/C |  |  |  |  |  |  |  |
|  | 3' UTR | c.*1067G>A |  | rs11628917 | G/A |  |  |  |  |  |  |  |
|  | 3' UTR | c.*1221A>G |  | rs11832 | G/G |  |  |  |  |  |  |  |
| 3 | Exon 1a | c.-535G>C |  | rs8008743 | G/C | MZ |  |  |  |  | 366Lys/366Glu(wt) |  |
|  | Exon 1a | c.-439C>T |  | rs8004738 | C/T |  |  |  |  |  |  |  |
|  | Exon 2d | c.-10T>C |  | rs11558258 | T/C |  |  |  |  |  |  |  |
|  | Exon 4 | c.710T>C | GTG>GCG p.Val237Ala | rs6647 | C/C |  |  |  |  |  |  |  |
|  | Exon 6 | c.1096G>A | GAG>AAG p.Glu366Lys | rs28929474 | G/A |  |  |  |  |  |  |  |
|  | 3' UTR | c.*80C>T |  | rs1243163 | C/T |  |  |  |  |  |  |  |
|  | 3´UTR | c.*316 G>T |  | rs1243164 | T/T |  |  |  |  |  |  |  |
|  | 3´UTR | c.*481G>A |  | rs1243165 | A/A |  |  |  |  |  |  |  |
|  | 3' UTR | c*854T>C |  | rs1051052 | T/C |  |  |  |  |  |  |  |
|  | 3' UTR | c.*968T>C |  | rs1243166 | C/C |  |  |  |  |  |  |  |
| 4 | Exon 1a | c.-409G>A |  | rs56367058 | G/A | MM |  |  |  |  |  | 400Asp/400Glu(wt) |
|  | Exon 2c | c.-10T>C |  | rs11558258 | T/C |  |  |  |  |  |  |  |
|  | Exon 6 | c.1200A>C | GAA>GAC p.Glu400Asp | rs1303 | A/C |  |  |  |  |  |  |  |
|  | 3' UTR | c.*224G>A |  |  | G/A |  |  |  |  |  |  |  |
|  | 3' UTR | c*854T>C |  | rs1051052 | T/C |  |  |  |  |  |  |  |
|  | 3' UTR | c.*968T>C |  | rs1243166 | T/C |  |  |  |  |  |  |  |
|  | 3' UTR | c.*1067G>A |  | rs11628917 | G/A |  |  |  |  |  |  |  |
|  | 3' UTR | c.*1221A>G |  | rs11832 | A/G |  |  |  |  |  |  |  |
| 5 | Exon 1a | c.-535G>C |  | rs8008743 | G/C | MZ | 125His/125Arg(wt) |  |  |  | 366Lys/366Glu(wt) | 400Asp/400Glu(wt) |
|  | Exon 1a | c.-439C>T |  | rs8004738 | C/T |  |  |  |  |  |  |  |
|  | Exon 3 | c.374G>A | CGT>CAT p.Arg125His | rs709932 | G/A |  |  |  |  |  |  |  |
|  | Exon 4 | c.710T>C | GTG>GCG p.Val237Ala | rs6647 | T/C |  |  |  |  |  |  |  |
|  | Exon 6 | c.1096G>A | GAG>AAG p.Glu366Lys | rs28929474 | A/G |  |  |  |  |  |  |  |
|  | Exon 6 | c.1200A>C | GAA>GAC p.Glu400Asp | rs1303 | A/C |  |  |  |  |  |  |  |
|  | 3' UTR | c.*224G>A |  |  | G/A |  |  |  |  |  |  |  |
|  | 3´UTR | c.*316 G>T |  | rs1243164 | G/T |  |  |  |  |  |  |  |
|  | 3´UTR | c.*481G>A |  | rs1243165 | G/A |  |  |  |  |  |  |  |
|  | 3' UTR | c.*968T>C |  | rs1243166 | C/C |  |  |  |  |  |  |  |
|  | 3' UTR | c.*1067G>A |  | rs11628917 | G/A |  |  |  |  |  |  |  |
|  | 3' UTR | c.*1221A>G |  | rs11832 | A/G |  |  |  |  |  |  |  |
| 6 | Exon 1a | c.-535G>C |  | rs8008743 | G/C | ZZ |  |  |  |  | 366Lys/366Lys |  |
|  | Exon 1a | c.-439C>T |  | rs8004738 | C/T |  |  |  |  |  |  |  |
|  | Exon 4 | c.710T>C | GTG>GCG p.Val237Ala | rs6647 | C/C |  |  |  |  |  |  |  |
|  | Exon 6 | c.1096G>A | GAG>AAG p.Glu366Lys | rs28929474 | A/A |  |  |  |  |  |  |  |
|  | 3´UTR | c.*316 G>T |  | rs1243164 | T/T |  |  |  |  |  |  |  |
|  | 3´UTR | c.*481G>A |  | rs1243165 | A/A |  |  |  |  |  |  |  |
|  | 3' UTR | c.*968T>C |  | rs1243166 | C/C |  |  |  |  |  |  |  |
| 7 | Exon 1a | c.-535G>C |  | rs8008743 | C/C (F) | ZZ |  |  |  |  | 366Lys/366Lys |  |
|  | Exon 1a | c.-439C>T |  | rs8004738 | T/T |  |  |  |  |  |  |  |
|  | Exon 4 | c.710T>C | GTG>GCG p.Val237Ala | rs6647 | C/C |  |  |  |  |  |  |  |
|  | Exon 6 | c.1096G>A | GAG>AAG p.Glu366Lys | rs28929474 | A/A |  |  |  |  |  |  |  |
|  | 3´UTR | c.*316 G>T |  | rs1243164 | T/T |  |  |  |  |  |  |  |
|  | 3´UTR | c.*481G>A |  | rs1243165 | A/A |  |  |  |  |  |  |  |
|  | 3' UTR | c.*968T>C |  | rs1243166 | C/C |  |  |  |  |  |  |  |
| 8 | Exon 1a | c.-535G>C |  | rs8008743 | G/C | MM |  |  |  |  |  |  |
|  | Exon 1a | c.-439C>T |  | rs8004738 | C/T |  |  |  |  |  |  |  |
|  | Exon 4 | c.710T>C | GTG>GCG p.Val237Ala | rs6647 | T/C |  |  |  |  |  |  |  |
|  | 3´UTR | c.*316 G>T |  | rs1243164 | G/T |  |  |  |  |  |  |  |
|  | 3´UTR | c.*481G>A |  | rs1243165 | G/A |  |  |  |  |  |  |  |
|  | 3' UTR | c*854T>C |  | rs1051052 | T/C |  |  |  |  |  |  |  |
|  | 3' UTR | c.*968T>C |  | rs1243166 | C/C |  |  |  |  |  |  |  |
|  | 3' UTR | c.*1221A>G |  | rs11832 | A/G |  |  |  |  |  |  |  |
| 9 | Exon 1a | c.-535G>C |  | rs8008743 | G/C | MZ |  |  |  |  | 366Lys/366Glu(wt) |  |
|  | Exon 1a | c.-439C>T |  | rs8004738 | C/T |  |  |  |  |  |  |  |
|  | Exon 4 | c.710T>C | GTG>GCG p.Val237Ala | rs6647 | T/C |  |  |  |  |  |  |  |
|  | Exon 6 | c.1096G>A | GAG>AAG p.Glu366Lys | rs28929474 | G/A |  |  |  |  |  |  |  |
|  | 3´UTR | c.*316 G>T |  | rs1243164 | G/T |  |  |  |  |  |  |  |
|  | 3´UTR | c.*481G>A |  | rs1243165 | G/A |  |  |  |  |  |  |  |
|  | 3' UTR | c*854T>C |  | rs1051052 | T/C |  |  |  |  |  |  |  |
|  | 3' UTR | c.*968T>C |  | rs1243166 | T/C |  |  |  |  |  |  |  |

| **#** | **Genotyping** | | | | | **IEF** | **LCMS** | | | | | |
| --- | --- | --- | --- | --- | --- | --- | --- | --- | --- | --- | --- | --- |
|  |  |  |  |  |  |  | **Deviations from wt-aa sequence mass** | | | | | |
|  | **Contig pos** | **HGVS** | **Amino acid change** | **Ref.** | **Result** | **Result** | **p.Arg125His** | **p.Glu280Val** | **p.Glu288Val** | **p.Ala308Ser** | **p.Glu366Lys (PiZ)** | **p.Glu400Asp (PiM2,M3)** |
|  | **SeqScape** | **position** |  |  |  |  | **(PiM2,M4)** | **(PiL_Lowell_)** | **(PiS)** |  |  |  |
| 10 | Exon 1a | c.-535G>C |  | rs8008743 | G/C | MM | 125His/125Arg(wt) |  |  |  |  | 400Asp/400Glu(wt) |
|  | Exon 1a | c.-439C>T |  | rs8004738 | C/T |  |  |  |  |  |  |  |
|  | Exon 3 | c.374G>A | CGT>CAT p.Arg125His | rs709932 | G/A |  |  |  |  |  |  |  |
|  | Exon 6 | c.1200A>C | GAA>GAC p.Glu400Asp | rs1303 | A/C |  |  |  |  |  |  |  |
|  | 3' UTR | c.*224G>A |  |  | G/A |  |  |  |  |  |  |  |
|  | 3' UTR | c.*968T>C |  | rs1243166 | T/C |  |  |  |  |  |  |  |
|  | 3' UTR | c.*1221A>G |  | rs11832 | A/G |  |  |  |  |  |  |  |
| 11 | Exon 1a | c.-535G>C |  | rs8008743 | G/C | MM | 125His/125Arg(wt) |  |  |  |  | 400Asp/400Glu(wt) |
|  | Exon 1a | c.-439C>T |  | rs8004738 | C/T |  |  |  |  |  |  |  |
|  | Exon 3 | c.374G>A | CGT>CAT p.Arg125His | rs709932 | G/A |  |  |  |  |  |  |  |
|  | Exon 6 | c.1200A>C | GAA>GAC p.Glu400Asp | rs1303 | A/C |  |  |  |  |  |  |  |
|  | 3' UTR | c.*224G>A |  |  | G/A |  |  |  |  |  |  |  |
|  | 3' UTR | c*854T>C |  | rs1051052 | T/C |  |  |  |  |  |  |  |
|  | 3' UTR | c.*1221A>G |  | rs11832 | A/G |  |  |  |  |  |  |  |
| 12 | Exon 1a | c.-535G>C |  | rs8008743 | C/C | ZZ |  |  |  |  | 366Lys/366Lys |  |
|  | Exon 1a | c.-439C>T |  | rs8004738 | T/T |  |  |  |  |  |  |  |
|  | Exon 4 | c.710T>C | GTG>GCG p.Val237Ala | rs6647 | C/C |  |  |  |  |  |  |  |
|  | Exon 6 | c.1096G>A | GAG>AAG p.Glu366Lys | rs28929474 | A/A |  |  |  |  |  |  |  |
|  | 3´UTR | c.*316 G>T |  | rs1243164 | T/T |  |  |  |  |  |  |  |
|  | 3´UTR | c.*481G>A |  | rs1243165 | A/A |  |  |  |  |  |  |  |
|  | 3' UTR | c.*968T>C |  | rs1243166 | C/C |  |  |  |  |  |  |  |
| 13 | 3' UTR | c*854T>C |  | rs1051052 | T/C | MM |  |  |  |  |  |  |
|  | 3' UTR | c.*968T>C |  | rs1243166 | T/C |  |  |  |  |  |  |  |
|  | 3' UTR | c.*1221A>G |  | rs11832 | A/G |  |  |  |  |  |  |  |
| 14 | 3' UTR | c*854T>C |  | rs1051052 | C/C | MM |  |  |  |  |  |  |
|  | 3' UTR | c.*968T>C |  | rs1243166 | T/C |  |  |  |  |  |  |  |
|  | 3' UTR | c.*1221A>G |  | rs11832 | A/G |  |  |  |  |  |  |  |
|  | 3' UTR | c.*1331G>A |  | rs11568814 | G/A |  |  |  |  |  |  |  |
| 15 | Exon 1a | c.-535G>C |  | rs8008743 | G/C | MM |  |  |  | 308Ser/308Ala(wt) |  | 400Asp/400Glu(wt) |
|  | Exon 1a | c.-439C>T |  | rs8004738 | C/T |  |  |  |  |  |  |  |
|  | Exon 4 | c.710T>C | GTG>GCG p.Val237Ala | rs6647 | T/C |  |  |  |  |  |  |  |
|  | Exon 5 | c.922G>T | GCC>TCC p.Ala308Ser | rs141620200 | G/T |  |  |  |  |  |  |  |
|  | Exon 6 | c.1200A>C | GAA>GAC p.Glu400Asp | rs1303 | A/C |  |  |  |  |  |  |  |
|  | 3' UTR | c.*80C>T |  | rs1243163 | C/T |  |  |  |  |  |  |  |
|  | 3' UTR | c.*224G>A |  |  | G/A |  |  |  |  |  |  |  |
|  | 3´UTR | c.*316 G>T |  | rs1243164 | G/T |  |  |  |  |  |  |  |
|  | 3´UTR | c.*481G>A |  | rs1243165 | G/A |  |  |  |  |  |  |  |
|  | 3' UTR | c*854T>C |  | rs1051052 | T/C |  |  |  |  |  |  |  |
|  | 3' UTR | c.*968T>C |  | rs1243166 | C/C |  |  |  |  |  |  |  |
|  | 3' UTR | c.*1067G>A |  | rs11628917 | G/A |  |  |  |  |  |  |  |
|  | 3' UTR | c.*1221A>G |  | rs11832 | A/G |  |  |  |  |  |  |  |
| 16 | Exon 1a | c.-535G>C |  | rs8008743 | G/C | MM |  |  |  |  |  |  |
|  | Exon 1a | c.-439C>T |  | rs8004738 | C/T |  |  |  |  |  |  |  |
|  | Exon 3 | c.374G>A | CGT>CAT p.Arg125His | rs709932 | G/A |  |  |  |  |  |  |  |
|  | Exon 6 | c.1200A>C | GAA>GAC p.Glu400Asp | rs1303 | A/C |  |  |  |  |  |  |  |
|  | 3' UTR | c.*224G>A |  |  | G/A |  |  |  |  |  |  |  |
|  | 3' UTR | c*854T>C |  | rs1051052 | T/C |  |  |  |  |  |  |  |
|  | 3' UTR | c.*968T>C |  | rs1243166 | C/C |  |  |  |  |  |  |  |
|  | 3' UTR | c.*1067G>A |  | rs11628917 | G/A |  |  |  |  |  |  |  |
|  | 3' UTR | c.*1221A>G |  | rs11832 | G/G |  |  |  |  |  |  |  |
|  | 3' UTR | c.*1331G>A |  | rs11568814 | G/A |  |  |  |  |  |  |  |
| 17 | Exon 4 | c.863A>T | GAA>GTA p.Glu288Val | rs17580 | T/T | SS |  |  | 288Val/288Val |  |  |  |
|  | 3’UTR | c.*854T>C |  | rs1051052 | C/C |  |  |  |  |  |  |  |
|  | 3’UTR | c.*968T>C |  | rs1243166 | C/C |  |  |  |  |  |  |  |
| 18 | Exon 4 | c.839A>T | GAT>GTT p.Asp280Val | rs121912714 | A/T | P_Lowell_S |  | 280Val/280Glu(wt) | 288Val/288Glu(wt) |  |  |  |
|  | Exon 4 | c.863A>T | GAA>GTA p-Glu288Val | rs17580 | A/T |  |  |  |  |  |  |  |
|  | 3’UTR | c.*854T>C |  | rs1051052 | C/C |  |  |  |  |  |  |  |
|  | 3’UTR | c.*968T>C |  | rs1243166 | C/C |  |  |  |  |  |  |  |

## Supplemental Table 3.

Correlation between phenotyping by IEF or phenotyping by LC-MS. 69/69 samples were concordant.

| **#** | **Genotyping** | | | | | **IEF** | **LCMS** | | | | | |
| --- | --- | --- | --- | --- | --- | --- | --- | --- | --- | --- | --- | --- |
|  |  |  |  |  |  |  | **Deviations from wt-aa sequence mass** | | | | | |
|  | **Contig pos** | **HGVS** | **Amino acid**  **change** | **Ref.** | **Result** | **Result** | **p.Arg125His** | **p.Glu280Val** | **p.Glu288Val** | **p.Ala308Ser** | **p.Glu366Lys (PiZ)** | **p.Glu400Asp (PiM2,M3)** |
|  | **SeqScape** | **position** |  |  |  |  | **(PiM2,M4)** | **(PiL_Lowell_)** | **(PiS)** |  |  |  |
| 19 |  |  |  |  |  | MM |  |  |  |  |  |  |
| 20 |  |  |  |  |  | MM |  |  |  |  |  |  |
| 21 |  |  |  |  |  | MZ |  |  |  |  | 366Lys/366Glu(wt) |  |
| 22 |  |  |  |  |  | SS |  |  | 288Val/288Val |  |  |  |
| 23 |  |  |  |  |  | SS |  |  | 288Val/288Val |  |  |  |
| 24 |  |  |  |  |  | SS |  |  | 288Val/288Val |  |  |  |
| 25 |  |  |  |  |  | MZ | 125His/125Arg(wt) |  |  |  | 366Lys/366Glu(wt) | 400Asp/400Glu(wt) |
| 26 |  |  |  |  |  | MZ | 125His/125Arg(wt) |  |  |  | 366Lys/366Glu(wt) | 400Asp/400Glu(wt) |
| 27 |  |  |  |  |  | SZ |  |  | 288Val/288Glu(wt) |  | 366Lys/366Glu(wt) |  |
| 28 |  |  |  |  |  | SZ |  |  | 288Val/288Glu(wt) |  | 366Lys/366Glu(wt) |  |
| 29 |  |  |  |  |  | MS |  |  | 288Val/288Glu(wt) |  |  | 400Asp/400Glu(wt) |
| 30 |  |  |  |  |  | MZ | 125His/125Arg(wt) |  |  |  | 366Lys/366Glu(wt) | 400Asp/400Glu(wt) |
| 31 |  |  |  |  |  | SZ |  |  | 288Val/288Glu(wt) |  | 366Lys/366Glu(wt) |  |
| 32 |  |  |  |  |  | FZ |  |  |  |  | 366Lys/366Glu(wt) |  |
| 33 |  |  |  |  |  | ZZ |  |  |  | 308Ser/308Ala(wt) | 366Lys/366Lys |  |
| 34 |  |  |  |  |  | MZ |  |  |  |  | 366Lys/366Glu(wt) |  |
| 35 |  |  |  |  |  | FF |  |  |  |  |  |  |
| 36 |  |  |  |  |  | MM | 125His/125Arg(wt) |  |  |  |  |  |
| 37 |  |  |  |  |  | MZ | 125His/125Arg(wt) |  |  |  | 366Lys/366Glu(wt) |  |
| 38 |  |  |  |  |  | MZ | 125His/125Arg(wt) |  |  |  | 366Lys/366Glu(wt) | 400Asp/400Glu(wt) |
| 39 |  |  |  |  |  | MZ |  |  |  |  | 366Lys/366Glu(wt) |  |
| 40 |  |  |  |  |  | MM |  |  |  |  |  |  |
| 41 |  |  |  |  |  | ZZ | 125His/125Arg(wt) |  |  |  | 366Lys/366Lys |  |
| 42 |  |  |  |  |  | MZ |  |  |  |  | 366Lys/366Glu(wt) |  |
| 43 |  |  |  |  |  | MZ |  |  |  |  | 366Lys/366Glu(wt) |  |
| 44 |  |  |  |  |  | MZ |  |  |  |  | 366Lys/366Glu(wt) |  |
| 45 |  |  |  |  |  | MM |  |  |  |  |  |  |
| 46 |  |  |  |  |  | MS |  |  | 288Val/288Glu(wt) |  |  |  |
| 47 |  |  |  |  |  | MZ |  |  |  |  | 366Lys/366Glu(wt) |  |
| 48 |  |  |  |  |  | MM |  |  |  |  |  |  |
| 49 |  |  |  |  |  | MM |  |  |  |  |  | 400Asp/400Glu(wt) |
| 50 |  |  |  |  |  | MM |  |  |  |  |  |  |
| 51 |  |  |  |  |  | SZ |  |  | 288Val/288Glu(wt) |  | 366Lys/366Glu(wt) |  |
| 52 |  |  |  |  |  | MZ |  |  |  |  | 366Lys/366Glu(wt) |  |
| 53 |  |  |  |  |  | MZ |  |  |  |  | 366Lys/366Glu(wt) |  |
| 54 |  |  |  |  |  | MM |  |  |  |  |  |  |
| 55 |  |  |  |  |  | MM | 125His/125Arg(wt) |  |  |  |  | 400Asp/400Glu(wt) |
| 56 |  |  |  |  |  | MM | 125His/125Arg(wt) |  |  |  |  | 400Asp/400Glu(wt) |
| 57 |  |  |  |  |  | MM | 125His/125Arg(wt) |  |  |  |  | 400Asp/400Glu(wt) |
| 58 |  |  |  |  |  | MM |  |  |  |  |  | 400Asp/400Glu(wt) |
| 59 |  |  |  |  |  | MM |  |  |  |  |  | 400Asp/400Glu(wt) |
| 60 |  |  |  |  |  | ZZ |  |  |  |  | 366Lys/366Lys |  |
| 61 |  |  |  |  |  | ZZ |  |  |  |  | 366Lys/366Lys |  |
| 62 |  |  |  |  |  | SZ |  |  | 288Val/288Glu(wt) |  | 366Lys/366Glu(wt) |  |
| 63 |  |  |  |  |  | MM |  |  |  |  |  |  |
| 64 |  |  |  |  |  | MM | 125His/125Arg(wt) |  |  |  |  | 400Asp/400Glu(wt) |
| 65 |  |  |  |  |  | MS | 125His/125Arg(wt) |  | 288Val/288Glu(wt) |  |  | 400Asp/400Glu(wt) |
| 66 |  |  |  |  |  | ZZ |  |  |  |  | 366Lys/366Lys |  |
| 67 |  |  |  |  |  | MM |  |  |  |  |  |  |
| 68 |  |  |  |  |  | MS |  |  | 288Val/288Glu(wt) |  |  |  |
| 69 |  |  |  |  |  | ZZ |  |  |  |  | 366Lys/366Lys |  |
| 70 |  |  |  |  |  | MS | 125His/125Arg(wt) |  | 288Val/288Glu(wt) |  |  | 400Asp/400Glu(wt) |
| 71 |  |  |  |  |  | MM |  |  |  |  |  |  |
| 72 |  |  |  |  |  | SZ |  |  | 288Val/288Glu(wt) |  | 366Lys/366Glu(wt) |  |
| 73 |  |  |  |  |  | MM |  |  |  |  |  | 400Asp/400Glu(wt) |
| 74 |  |  |  |  |  | ZZ |  |  |  |  | 366Lys/366Lys |  |
| 75 |  |  |  |  |  | MZ |  |  |  |  | 366Lys/366Glu(wt) |  |
| 76 |  |  |  |  |  | MM |  |  |  |  |  |  |
| 77 |  |  |  |  |  | MZ |  |  |  |  | 366Lys/366Glu(wt) |  |
| 78 |  |  |  |  |  | MM |  |  |  |  |  |  |
| 79 |  |  |  |  |  | SZ |  |  | 288Val/288Glu(wt) |  | 366Lys/366Glu(wt) |  |
| 80 |  |  |  |  |  | ZZ |  |  |  |  | 366Lys/366Lys |  |
| 81 |  |  |  |  |  | MM |  |  |  |  |  |  |
| 82 |  |  |  |  |  | MM |  |  |  |  |  |  |
| 83 |  |  |  |  |  | MM |  |  |  |  |  |  |
| 84 |  |  |  |  |  | MM |  |  |  |  |  |  |
